# Supplementary material for: Uptake of community antiretroviral group delivery models for persons living with HIV in Arua district, Uganda: A parallel convergent mixed methods study
Source: PLOS Glob Public Health. 2023 Feb 23;3(2):e0000633. doi: 10.1371/journal.pgph.0000633 (PMC10021482; doi:10.1371/journal.pgph.0000633)
Supplement: Attachment — Submitted filename: Response to reviwers.docx [file pgph.0000633.s002.docx]

Journal requirements

1. Copy edit

Response

2. Additional details regarding participant consent

Response: this has been revised under: under ethics approval and consent to participate

3. Data availability

Response: Both qualitative and quantitative data sets have been uploaded as supplementary files

Reviewer 1

1. Data availability:

Response: Both qualitative and quantitative data has been uploaded as supplementary files. The data availability statement has been revised.

2. Other points of contact

Response: Research ethics committee email address has been under ethics and consent to participate.

3. Typos and informal language:

Response: The manuscript has been edited by a professional English language editor

4. Acronyms should be written out fully when first used in the text:

Response: This has been corrected in the abstract.

5. Keywords left blank

Response: This has been corrected and key words have been added

6. Introduction

Response: The introduction has been revised

7. Methods

Response: The methods have been revised accordingly

8. Results

Response: The results have been revised accordingly

9. Discussion

Response: The discussion has been revised.

Reviewer 2

1. Research question. The research question has been revised
2. Justification of mixed methods. Mixing of the methods was done in the discussion and the justification has been provided.(see study design)
3. Justification of FGDs. This has been included(see data collection under qualitative)
4. Context. The context has been revised.
5. Eligibility. This has been revised(see study population and paragraph 3 of introduction)
6. Qualitative data. This has been revised accordingly(see appropriate heading)
7. Data extraction. This has been revised
8. Results: This has been revised accordingly
9. Discussion: Discussion has been revised accordingly
